# Complexity of Nurse Practitioners’ Role in Facilitating a Dignified Death for Long-Term Care Home Residents during the COVID-19 Pandemic

**DOI:** 10.3390/jpm11050433

**Published:** 2021-05-19

**Authors:** Shirin Vellani, Veronique Boscart, Astrid Escrig-Pinol, Alexia Cumal, Alexandra Krassikova, Souraya Sidani, Nancy Zheng, Lydia Yeung, Katherine S. McGilton

**Affiliations:** 1 KITE, Toronto Rehabilitation Institute–University Health Network, Toronto, ON M5G 2A2, Canada; shirin.vellani@mail.utoronto.ca (S.V.); vboscart@conestogac.on.ca (V.B.); aescrig@esimar.edu.es (A.E.-P.); alexia.cumal@mail.utoronto.ca (A.C.); alexandra.krassikova@mail.utoronto.ca (A.K.); nancy.zheng@mail.utoronto.ca (N.Z.); Lydia.yeung@uhn.ca (L.Y.); 2 Lawrence S. Bloomberg, Faculty of Nursing, University of Toronto, Toronto, ON M5T 1P8, Canada; 3 Canadian Institute for Seniors Care, Conestoga College, Kitchener, ON N2G 4M4, Canada; 4 Mar Nursing School, Universitat Pompeu Fabra, 08002 Barcelona, Spain; 5 Rehabilitation Sciences Institute, Faculty of Medicine, University of Toronto, Toronto, ON M5G 1V7, Canada; 6 Daphne Cockwell School of Nursing, Ryerson University, Toronto, ON M5B 1Z5, Canada; ssidani@ryerson.ca

**Keywords:** nurse practitioners, nursing home, COVID-19, palliative care, end-of-life, dignified death, older adults

## Abstract

Due to the interplay of multiple complex and interrelated factors, long-term care (LTC) home residents are increasingly vulnerable to sustaining poor outcomes in crisis situations such as the COVID-19 pandemic. While death is considered an unavoidable end for LTC home residents, the importance of facilitating a good death is one of the primary goals of palliative and end-of-life care. Nurse practitioners (NPs) are well-situated to optimize the palliative and end-of-life care needs of LTC home residents. This study explores the role of NPs in facilitating a dignified death for LTC home residents while also facing increased pressures related to the COVID-19 pandemic. The current exploratory qualitative study employed a phenomenological approach. A purposive sample of 14 NPs working in LTC homes was recruited. Data were generated using semi-structured interviews and examined using thematic analysis. Three categories were derived: (a) advance care planning and goals of care discussions; (b) pain and symptom management at the end-of-life; and (c) care after death. The findings suggest that further implementation of the NP role in LTC homes in collaboration with LTC home team and external partners will promote a good death and optimize the experiences of residents and their care partners during the end-of-life journey.

## 1. Introduction

Individuals transition into long-term care (LTC) homes closer to the end of their life, often with multiple comorbid conditions, higher levels of frailty and complex care needs [1,2]. Within two years of admittance to LTC homes, most residents die [3,4]. As such, integrating a palliative approach to care would be the best practice, yet this approach is not implemented in the majority of LTC homes [5]. A palliative approach consists of four central components: advance care planning (ACP); optimization of pain and symptom management; psychosocial and spiritual support for patients and their care partners such as friends and relatives; as well as shared decision-making [6]. While death is an unavoidable ending for LTC residents, promoting a good death is an important goal of palliative and end-of-life (EOL) care [7,8]. This has become even more pressing during the SARS Coronavirus Disease-2019 (COVID-19) pandemic, where a large percentage of deaths were among LTC home residents, ranging from 8% in South Korea, 39% in the United States [9] to 69% in Canada [10]. A good death is characterized by freedom from preventable distress in a dying person, their care partners, and healthcare providers; being in harmony with the wishes of the dying person and their family; and following clinical, cultural, and ethical principles [8].

Sufficient staffing is critical for integrating a palliative approach to care and to achieve a good death in LTC settings [11]. However, the LTC sector continues to suffer from chronic staff shortages. Furthermore, to optimize the infection prevention and control measures, LTC home residents have been forced to isolate in their own spaces with visitation restrictions from informal care partners [12]. Traditionally, care partners served as a means for socialization and comfort for residents and also assisted them with activities of daily living [13] and EOL care. During the pandemic, there was also a shift in physicians’ presence in LTC homes, where many only worked virtually because of their multiple responsibilities across different sites [14], limiting the planning and implementation of palliative care. In contrast, nurse practitioners (NPs) in Ontario were able to work on-site [15] and provide palliative and EOL care by functioning closely with the registered nurses, registered practical nurses, and personal support workers in LTC homes [16]. In Ontario, Canada, NPs are advanced practice registered nurses, who can autonomously assess, diagnose, and treat patients [17,18] and are well situated to optimize the palliative and EOL care needs of LTC home residents. Empirical evidence demonstrates that palliative care provided by NPs in various settings was effective in improving persons’ emotional and mental wellbeing as well as their quality of life [19]. NPs also increase ACP [20,21,22] and provide effective management of EOL symptoms [23,24].

There is limited knowledge of the role of NPs in delivering and coordinating EOL care and facilitating a “dignified death” for LTC home residents during the COVID-19 pandemic. The term “dignified death” is used in this study as the pandemic has hampered the staff’s ability to ensure a good death by exposing the entire LTC sector to a myriad of challenges such as understaffing, limited family contacts and a lack of palliative care supplies in some LTC homes [25,26]. In this study, we focused on NPs’ perspectives and aimed to explore their role and experiences in facilitating a dignified death for LTC home residents, while also facing the increased pressures related to the COVID-19 pandemic. The findings will provide more insight and understanding into the roles NPs can play in integrating a palliative approach to care in LTC settings. Additionally, they will highlight knowledge gaps in optimizing resident-centered palliative care in LTC homes and yield implications for policy makers, researchers and clinicians.

## 2. Methods

### 2.1. Study Design and Participants

We designed an exploratory qualitative study to examine the role and experiences of NPs in supporting a dignified death during the COVID-19 pandemic, as the phenomenon is not yet well-observed or understood [27]. The study used a phenomenological approach, which is valuable in revealing shared and divergent experiences among participants [28]. We conducted telephone-based semi-structured interviews with NPs providing care to LTC home residents during the pandemic. Recruitment of NPs was assisted by the Nurse Practitioners Association of Ontario (npao.org). An email with information about the project, inclusion criteria, and an invitation to connect with the Research Coordinator (RC) was sent out to all NPAO members by their Practice and Policy Manager. The inclusion criteria were NPs who worked at least three days a week in an LTC home that had experienced positive cases of COVID-19.

The final sample included 14 NPs from 14 separate LTC homes, representing approximately 13% of all NPs working in the province of Ontario LTC home sector. The recruited NPs worked in both rural and urban settings, as well as in an even mix of profit and not-for-profit homes. This sample size was determined by reaching data saturation, i.e., information became repetitious from one interview to the next [29]. The characteristics of the participating NPs and their respective LTC homes are summarized in Table 1.

Most participants were employed full-time and experienced an increase in work hours during the pandemic. Most NPs were females, with an average of nine years of experience in the NP role. Eight were attending NPs and six were outreach NPs. Attending NPs provide direct primary health care to residents, work to increase the knowledge and skills of LTC staff and participate in administrative and leadership activities to improve resident outcomes and reduce pressures on acute care services [30]. The outreach NP role was created to provide comprehensive acute and episodic care to LTC home residents in order to avoid transfers to the emergency department, facilitate hospital transfers where necessary and reduce hospital stays through early repatriation back to LTC homes [31].

### 2.2. Procedures

After receiving approval from the Research Ethics Board at the participating institution, we obtained informed written consent from participants. In order to maintain their privacy, each participant was assigned a unique identification number and all their identifying information (e.g., name of the LTC home) was anonymized in the interview transcripts. Furthermore, the research team had considered potential emotional distress for the participants with sharing their experiences during the pandemic. Therefore, participants were offered breaks, opportunity to reschedule or drop-out and resources for emotional support during the interview, when appropriate. Telephone interviews were conducted by the RC (AK), while a note-taker (LY or AC) recorded the topics conversed and other relevant information. We audio-recorded the interviews, which, on average, lasted 50 min and took place between August and October 2020. Participants were provided a CAD 50 gift card as an honorarium serving as a token of appreciation for their time and effort to participate during these extremely busy and challenging times imposed by the pandemic. Most NPs participated in the study after their regular work hours to prevent any work-time lost deserving recognition from the research team and the honorarium may have served as an incentive to participate. However, gift card was only introduced after NPs had expressed desire to participate to prevent the risk of undue inducement as suggested by other authors [32]. Additionally, participants had the option of leaving the study at any time.

Semi-structured interviews were designed to prompt NPs to elaborate and reflect on their experiences [33] related to EOL care before and during the pandemic, their thoughts on their roles and responsibilities, and how they adapted in the context of the crisis. The interview guide was piloted for clarity and relevance with an NP experienced in LTC homes, but this was not included as a part of the study. The interview guide is in Supplementary Material Table S1.

### 2.3. Data Analysis

The RC reviewed and anonymized the professionally transcribed interviews by using participant ID numbers, guaranteeing confidentiality. Data were exported into NVIVO12 to be managed, organized, and coded. This provided the research team a greater degree of transparency and integrity as members were able to create notes on their thoughts alongside the participant data. It also allowed for tracking the analysis process efficiently and mapping the relationship between data. However, some team members had no prior experience of using the NVIVO software, which required more time to train members on its features before starting the analysis process. There were also issues with software compatibility with different operating systems.

We employed an inductive thematic analysis strategy adapted from Braun and Clark [34]. The analysis was conducted in five steps. The first step involved familiarization with the data, which involved the research team reading the interview transcripts multiple times and writing down ideas as they arose in the due process. Debrief sessions were held after each interview to discuss and record emerging topics. In the second step, the initial themes were generated in a systematic manner across all the interviews. Two analysts (a primary analyst (AK) and a secondary analyst (either SV/AC/LY/NZ)) independently coded each transcript into initial themes and met regularly to compare and reconcile coding divergences. The third step involved the identification of broader categories by collating the codes and gathering data appropriate to each category. Preliminary themes were used as a point of departure and evolved as analysts generated additional themes during the coding stage. We organized these topics into 10 preliminary themes. Initial themes were grouped into three broad categories. In the fourth stage, broader categories were reviewed by the full research team to reach a consensus on these categories. Once the consensus was reached, the analysis team verified the coherence of categories and themes by reading all collated extracts for each theme, while also confirming that they were sufficiently and meaningfully different from each other [34]. This strategy allowed for refinement of the themes and categories. In the final step, the research team generated clear definitions and names of the categories and themes [34,35], as presented in Table 2 below.

Credibility was ensured by conducting systematic peer debriefing sessions, incorporating researcher reflexivity and triangulation, keeping an exhaustive record of the process and decisions made, managing data systematically, and analyzing opposing accounts [33,36]. Discussions during these peer debriefing sessions resulted in adjustments in the order and phrasing of questions to improve the flow and clarity during subsequent interviews, and in a list of preliminary themes appearing on each interview. For example, while we initially thought there was a sub-category related to how NPs partnered with families to ensure a dignified death, with more debriefing sessions and subsequent interviews, we realized that NPs reached out to family members for different purposes along the journey. Thus, the specific reasons for reaching out to families were included as themes within each category to better represent the important role NPs had related to connecting with families in all aspects of EOL care. The Standards for Reporting Qualitative Research guidelines were followed [37].

## 3. Results

Three categories were derived in relation to the NPs’ roles and responsibilities in facilitating a dignified death for LTC home residents under their care: (a) ACP and goals of care discussions; (b) pain and symptom management at the EOL; and (c) care after death. The categories are presented in Table 2 and detailed below.

### 3.1. ACP and Goals of Care Discussions

NPs described this responsibility as engaging with residents and more frequently with their health care proxies, who were often care partners, by taking a proactive approach to facilitate ACP and goals of care conversations. They connected with care partners for difficult, yet critical, discussions when the residents appeared to be nearing the EOL. During the pandemic, these conversations with care partners usually happened virtually over the telephone or through video calls. NPs attested to the importance of implementing a palliative approach to care where the focus is on the residents’ quality of life, while also adhering to the safety requirements necessitated by the COVID-19 pandemic. Many highlighted the complexity of these discussions in light of care partners’ inability to see firsthand the residents’ decline due to the physical distancing restrictions. One NP explained:

*This was time-consuming, but we were very successful in bringing families into sort of an understanding of reality and acceptance of end-of-life issues… advance-care planning was a huge, huge part of my time–helping families to move along that continuum.*

(NP 13)

NPs explained that, generally, ACP and goals of care conversations are carried out with care partners and residents at the time of admission to the LTC home, and regularly thereafter, particularly when any acute changes in condition arise. However, during the pandemic, the need and frequency of these conversations increased markedly, as explained by this NP:

*If there’s a change in the resident’s condition, I would be the one in touch with the families. It was a bit more fast-forwarded (during the pandemic). I just had so many of them. So, I would have daily conversations with probably five to 10 families about goals of care.*

(NP 3)

Some LTC homes did not have the resources to tackle these discussions in a timely way. Therefore, several NP participants were specifically assigned by their LTC home management to support LTC staff in holding ACP and goals of care discussions with families, given the constant risk of a sudden decline in residents’ condition. In addition, several NPs organized and chaired virtual care conferences linking families with the care team and physicians, involving residents whenever possible. One NP (*NP 8*) noted that their team was called “palliative rescue”, alluding to the fact that when there was a COVID-19 outbreak, several residents became sick very fast, potentially needing life-sustaining treatments and goals of care discussions. This NP explained:

*One of my homes I was supporting virtually and via telephone had a COVID outbreak. They started with 10 cases, and then within three days, they had 30, and then it was escalating, and then by the end of the week, they had over 77 cases. So, at that point, we spoke with the DOC [director of care] and were asked to support goals of care discussions with the families because the home had not done them and so the entire outreach team started calling families to discuss goals of care.*

(NP 8)

Many NPs noted that communication with families was a responsibility that frequently fell upon them during the pandemic. Several NPs indicated that although these conversations were lengthy, they were gratifying. NPs engaged in difficult conversations with care partners regarding prior requests for life-sustaining treatments, such as resuscitation, in light of the inherent risk of aerosolizing COVID-19 viral particles during such interventions, and the impact of these treatments on residents’ quality of life and wellbeing. These interactions provided care partners the opportunity for shared decision-making to devise a plan of care, whether that involved comfort care in the LTC home or transfer to a hospital, which was not always possible due to the pandemic and restrictions imposed by the acute care hospitals, making these conversations even more difficult. In terms of restrictions, some NPs indicated that in order to prepare for the surge of patients with COVID-19 in acute care, some administrators within LTC homes were informed to avoid hospital transfers by managing their residents’ acute changes in condition within their homes. As such, NPs needed to make decisions on how to balance the needs of the residents and potential outcomes of life-sustaining treatments. Some NPs made these difficult decisions in consultation with emergency department physicians, which was often made possible based on a history of working together.

NPs were sensitive to the fact that care partners could not be present physically due to the social distancing measures when the residents were dying. As such, in many cases, NPs helped family members appreciate the inevitable nearing of the EOL and also provided much needed emotional support. One participant explained:

*Yes, communicating with families was my big responsibility really. It sort of just fell upon me, explaining to the families what was happening and if it looked like the person was at the end-of-life, then I would be talking to them about palliative care and what things we could do for the person in the home in order for them to have a dignified death in the home.*

(NP 12)

Overall, NPs made it their duty to keep the families abreast of the residents’ condition, such as how COVID-19 was affecting them and what the care options were for the day, which served as a lifeline for the families given their inability to be physically present with the resident. They served as a link between the family and the residents, listened to the families’ fears and concerns, and answered their questions.

### 3.2. Pain and Symptom Management at the EOL

The NPs’ second responsibility consisted of optimizing comfort for residents who were at the EOL and where death was imminent, through pain and symptom management. This involved working closely with LTC home staff and management teams in optimizing emergency supplies and planning ahead for emergencies; prescribing anticipatory medications to aid EOL symptom management; consulting with expert clinicians where needed; and addressing the psychosocial needs of residents and families. Some NPs played an instrumental role in creating the EOL order sets with input from palliative care physicians in their local hospital. They also updated these order sets regularly to include relevant symptom management for residents dying of COVID-19 infection due to its unique symptom profile because the appearance and progression of symptoms such as respiratory distress can be rapid and fulminant, intensifying over a short period of time; and shared them with clinicians at other LTC homes. Additionally, NPs led educational initiatives to prepare and guide staff on interventions pertaining to intravenous therapy and hypodermoclysis. Moreover, NPs became involved in sourcing an optimal stock of emergency supplies such as oxygen, parenteral antibiotics and analgesics in the event that they were needed after hours. One NP explained:

*We had a lot more End-of-Life meds in the building. We had more oxygen concentrators. We had more IM antibiotics, more Hydromorphone…we did increase our emergency stock med, so that we could…like at 2:00 a.m. if we needed to. So, I guess that’s the other thing we did plan.*

(NP 6)

In general, NPs were instrumental in ensuring that analgesics and other medications were available when needed for palliative and EOL care needs. Many NPs stressed the importance of collaborative teamwork involving LTC home nurses and other staff to optimize residents’ care given the scarcity of resources and shortage of staff.

NPs expressed that it was difficult to complete timely in-person assessments given the challenges associated with insufficient staffing and the time required to don and doff the personal protective equipment (PPE) with each resident interaction. Despite this, they tried their best and worked with other staff for ongoing identification of distressing symptoms, prescribing anticipatory and emergent treatments to address them, specifically in cases where EOL order sets had not been implemented. Many referred to the fact that LTC home physicians were generally not present in person during the pandemic, requiring NPs to manage this limitation in medical management. Therefore, NPs also assisted staff in the safe and timely delivery and assessment of prescribed therapies, including opioids. Ultimately, together, the teams ensured that residents died peacefully. Several NPs expressed that EOL care was most challenging because of the physical/social isolation, whereby direct contact with residents was more limited, in addition to family and friends not being able to visit. Many NPs found that working closely with the LTC staff and connecting with other NPs working in the LTC sector served as sources of strength to deal with challenges they experienced during these times. NPs ensured that staff and residents’ care partners had their direct numbers to reach out any time of the day. Moreover, they worked hard to identify residents close to the EOL and ensured that symptom management was provided, as this quote exemplifies:

*We just really wanted to identify the residents who were unfortunately really sick or passing away from COVID. Because we didn’t want them to die uncomfortably and alone and without any support and care. And that was probably the most emotional because you find residents with a respiratory rate of 50 and they’re diaphoretic and they’re struggling to breathe and they’re alone. And so, we did provide our assessments and we tried to give the residents the treatment that they needed to die comfortably.*

(NP 10)

NPs talked about the complexity of LTC home resident care associated with their advanced age and multimorbidities, sometimes requiring consultation with expert clinicians and services to optimize pain and symptom management at the EOL. NPs consulted physicians with expertise in nephrology, palliative care, and geriatrics mostly virtually when they were unable to visit in-person. Furthermore, to address situations when they were not available, NPs developed algorithms for LTC home staff on how to consult with specialists. In some LTC homes, palliative care physicians provided expert consultations to NPs in order to facilitate a dignified death for residents with more complex needs, as is highlighted in this quote:

*So, the ten residents that died, I provided their palliative care, their end-of-life symptom management. And if there was a resident that I was having difficulty managing their symptoms, I would call and consult with a palliative care physician to get those symptoms under control.*

(NP 1)

Therefore, NPs’ resourcefulness in consulting with clinical experts helped to ensure the better control of symptoms for residents who were nearing death.

Many NPs also addressed the psychosocial needs of residents and their families, particularly when the residents were nearing the EOL. They expressed that sitting vigil at the time of death is considered a norm in LTC homes; however, this was challenging during the pandemic. They felt a sense of responsibility to promote human connection in these difficult circumstances as best as possible, through creative means, with support from LTC home staff. Several NPs pointed out that compassionate visits were offered to care partners when their residents were imminently dying, allowing them to stay as long as possible while making sure that their PPE remained safely usable. This was illustrated by NPs in the following quotes:

*When we were talking about comfort or end-of-life, then we were offering compassionate visits. So essentially once I was in contact with the families, then I would put them on the list of allowed visitors.*

(NP 3)

*As soon as we possibly could, if people were dying, knowing they were clearly at the end-of-life, then families could come in–one person at a time with full PPE.*

(NP 13)

In cases where families could come but were unable to be at the bedside due to the active risk of exposure to the aerosolized virus, NPs and LTC home staff tried unprecedented and creative ways to allow families and residents to see and hear each other through a window or virtual means. One NP shared her experience with a resident that involved juggling with safety procedures while also enabling family connection in the last moments of life:

*I feel at peace at least with what we were able to do for her… her oxygen requirements were going up fast …potentially aerosolizing the COVID virus. … we knew she was going to pass away from COVID … and she was on the main floor so the family was able to come to her window. We were able to sort of set up the phone on our end and then call their cell phone on the other side, so it kind of looked like you were talking to each other … it was very sad but it was also, you know, the quote “good death” if you will.*

(NP 5)

Finding creative ways to promote connection and support the psychological needs of the resident and care partners at the last moments of life highlights the critical role the NPs played.

Moreover, in many cases, NPs collaborated with LTC home staff such as personal support workers to keep vigil at the time of residents’ death so that they would not die alone. They held residents’ hands or played a musical instrument when the care partner could not be at their bedside. This was believed to be a source of comfort for residents as well as staff, and in line with enabling a dignified death given the restrictions imposed by the COVID-19 pandemic.

### 3.3. Care after Death

The third NP role responsibility involved being present with the staff for the dignified performance of last offices and providing emotional support to staff and care partners upon residents’ death. Care after death also included informing the care partners of the death, upholding the pandemic-related policy of allowing only one grieving care partner at a time, and completing death certificates, sometimes after hours. Several NPs expressed feeling significant moral distress as the pandemic led to losing several residents under their care in a short period of time, in addition to seeing care partners lose time for genuine human connections that will not be replaced. One NP explained:

*It was very difficult to watch families come in one at a time and try to manage their grieving by themselves, with their other family in the parking lot. That’s what’s changed at end-of-life; that’s what made it so difficult. Calling a resident’s family to say that they passed away, and they weren’t there.*

(NP 1)

Despite their own high emotional burden, NPs worked hard to provide emotional support to grieving care partners.

NPs also worked closely with LTC home nurses and the administrators in devising and implementing new policies related to the care after death. To contain the potential spread of the COVID-19 virus, they had a very restricted amount of time to pronounce death, inform the family, perform the last offices (previously performed by the funeral homes), and hand over the body to the funeral home staff, who were not allowed to come into the building; all of this while avoiding the omnipresent media. NPs identified that they were under a great deal of pressure to prevent the accumulation of bodies in LTC homes. NPs took on the leadership role in identifying the best practices in performing the last offices with dignity and care as well as instituting specific considerations for those who died with COVID-19 infection. They educated staff on COVID-19-focused care after death through demonstrations and hands-on help. As, many unregistered staff had no previous experience of performing last offices. One NP describes their post-death experience as follows:

*The other thing that we had a lot of policy around, this sounds horrible, but on pronouncing death and removing bodies from the home. This was a nightmare. We got this thing that said nobody could come in the home to take them out—I’m going to cry…but we had to do all of the post-mortem care and the nurses found that so hard. So again, we needed to look at how it was done, how do you transport people out. How do we keep the media from photographing people that died as they’re being taken out of the home? It was brutal.*

(NP 13)

Given that NPs in the province of Ontario have the ability to complete the death certificates if they had the primary responsibility of the patient, they were responsible for completing them as soon as the residents passed. This NP describes her experience below:

*All the death certificates were completed online. I would often get a phone call in the middle of the night to do a death certificate, like at 2:00 a.m. to complete one, where those could have waited ‘till the next morning, previously.*

(NP 6)

As such, NPs worked beyond normal hours to fulfill their duties that also involved administrative tasks post residents’ death, highlighting the significance of their role and the added stress.

NPs described challenges encountered by the LTC home staff after any resident’s death due to experiencing pandemic-based disenfranchised grief. For example, novel and continuously changing COVID-19 policies affected how LTC home staff would traditionally perform care after death. As a result, NPs had to meet the pandemic-imposed demands while also mindfully allocating large amounts of time and effort in working closely with nurses and other staff to adapt new policies and providing them emotional support to recognize and process the losses. One participant stated:

*Because normally the way nursing homes deal with resident death is the funeral home would come and prepare the body and whatnot. And there’s actually a ceremony, not a real ceremony, but all the staff lines up at the front entrance and it’s more of a respectful send-off to the resident, whereas this is kind of like “OK, let’s just get the body ready and take them outside.” And no one was allowed to be there to witness all of this stuff. So, it just doesn’t feel as humanistic as how it was done pre-COVID.*

(NP 2)

NPs provided emotional support to staff, especially after the changes to the processes after death were made, which often left little room for staff’s own grieving. As one NP noted:

*What I learned very quickly is she [staff] just needed to be listened to, and just needed to have someone who could validate her fears and say yeah, we don’t know all the answers but we’re going to get through this, and we’re going to do it together… mostly it was listening, listening, listening, and modeling. And trying–and not appearing fearful yourself.*

(NP 13)

Many NPs expressed that given the challenges associated with working during the pandemic, they had minimal time to focus on in their own self-care; however, they found communication exchanges with other staff helpful. NPs relayed a sense of accomplishment and gratitude to be a part of the healthcare workforce during this pandemic. They highlighted that their work was complemented by the LTC home staff and administrators, all of whom were devoted to doing their best to provide person-centered care during this time of crisis when much was unknown. It appears that NPs served as a binding force that played a crucial role in holding together the staff and residents in facilitating a dignified death for the residents.

## 4. Discussion

SARS COVID-19-related adverse events and mortality have been the highest in the LTC sector globally [38,39]. This is because of a complex interplay of multiple intricate and inextricably connected factors that increase the vulnerability of LTC home residents to not only contract COVID-19 infection but also to sustain poor outcomes, including death [40,41]. Amidst this unprecedented crisis, there has been a further decline in already limited human and material resources in LTC homes to optimally provide for the needs of the residents and the workforce, who continued to care for their residents with threadbare resources [42].

Residents admitted to LTC homes are increasingly complex, with high care needs [1,2]. Death is a critical juncture for not only the residents but also their care partners and LTC home staff [3,4]. However, the COVID-19 pandemic brought a variety of challenges in the achievement of a good death. This study demonstrated that NPs, along with staff within LTC home teams, collaborated and worked tirelessly to support a dignified death experience for residents. NPs and staff assisted in ACP and goals of care discussions with residents and care partners, both proactively and when residents showed signs of acute changes in condition. This, in turn, helped care partners move forward in accepting the impending EOL. Findings from our study correlate with other studies that demonstrated an increase in ACP discussion where NPs were involved in the residents’ care team [20,21,22], with one study showing a 300% increase in the number of residents with ACP discussions [20]. Similar to the findings in our study, Campbell and colleagues also found that NPs were part of the collective approach in ensuring residents were frequently assessed, especially those in the last stage of their life [43]. NPs worked hard so that residents’ distressing symptoms were addressed, that they were comfortable, and died in their familiar surroundings rather than a hospital, when appropriate. NPs helped LTC home staff and administrators implement new policies and procedures related to post-death care, such as dignified care of the deceased, while maintaining the infection prevention and control measures, in addition to catering to the emotional needs of the staff and grieving care partners.

Our study demonstrated that NPs played a vital role in promoting a dignified death for LTC home residents while navigating the challenges of the COVID-19 pandemic. Based on previous studies, a good death is a broad construct where multiple factors can play a role to achieve it [44]. These factors can be related to the individual experiencing the death, their care partners as well as healthcare providers. Allison and O’Connor describe a 6-step framework used by clinical nurse specialists to facilitate a good death in residential aged care facilities [45]. The framework involved (1) responding within 24–48 h of a referral; (2) visiting the residential aged care setting to assess the resident and train the staff; (3) developing a care plan; (4) encouraging staff to proactively request medications for pain, respiratory secretion, and nausea from the general practitioner; (5) liaising with management and ensuring support for staff; and (6) involving the family in care planning and preparing them for death. The findings from our study suggest that NPs were able to faciliate a dignified death which appeared to be aligned with the 6-step framework provided by Allison and O’Connor, but also did more due to their expanded scope of practice. For example, some NPs were available 24 h a day either in person or over the phone, not only to staff but also to residents’ care partners. Additionally, they were able to prescribe medications including opioids in order to maximize comfort in the last moments of life.

In addition to pain control, peace, and dignity, the presence of care partners, and being surrounded by familiar people and things have also been identified as sources promoting a good death in those dying with dementia [46]. Though people’s preferences for the presence of others may vary, having someone present at the time of death can serve to address the primal need for companionship and is seen as a closing chance to display comfort and affection to the resident [47]. Whereas COVID-19 stripped the care partners of these opportunities, NPs collaborated with LTC home staff in identifying ways to connect them through virtual and other creative means. NPs or the staff also sat vigil by the residents’ bedside of those who were in the last moments of their life. However, there remains a need for further initiatives and research on how to best care for residents’ spiritual and cultural needs as well as the needs of the bereaved in light of the strange circumstances imposed by the COVID-19 pandemic [48].

This study highlights several research, policy, and clinical implications. NPs fostered interdisciplinary collaboration for in-house care that embraced an integrated palliative approach with chronic disease management. However, a palliative approach to care is not a norm in the LTC sector and regular ACP discussions are not a standard of practice [49]. ACP discussions are generally held in the form of level of care documentation to identify residents’ and their proxy’s wishes related to hospital admission and resuscitation at the time of admission to the LTC home and do not necessarily identify their values and wishes such as for their EOL care. Furthermore, previous research has identified that non-palliative specialist NPs who have provided palliative care to people under their care have also expressed the need for education in palliative care [50], highlighting a gap in relevant training and education. A recent work commissioned by the Alzheimer Society of Canada on Improving End-of-Life Care for People with Dementia in LTC homes during the COVID-19 pandemic and beyond proposes three strategies to implement a palliative approach [49]. These include (a) adopting it in the whole home; (b) education and training of all staff on a palliative approach to care; and (c) implementing policies and tools that assist staff to use their knowledge about a palliative approach to enhance resident care [49]. Some LTC homes have successfully implemented a palliative approach through initiatives such as the Strengthening a Palliative Approach in Long Term Care Model (SPA-LTC) [51,52]. Hence, there is much to learn from their experiences in order to successfully implement this approach in other homes and to increase the capacity of LTC home staff, including the NPs as well as external consultants. Additionally, there is a need to implement the same palliative care standards of practice across all practice settings, appreciating the highly complex resident population served by LTC homes.

NPs working collaboratively with their team members have demonstrated the ability to provide high quality palliative and EOL care [19,23,53]. Countries and regions where the role of NPs is either non-existent or in infancy can learn from the experiences of various regions in Canada, the USA and the UK, where the role of the NP has been successfully implemented [18] to be able to autonomously assess, diagnose, order, and interpret diagnostic tests and treat their patients with full prescriptive authority. In addition, they are able to collaborate with other healthcare practitioners including primary care physicians and palliative care specialists and seek further advice when required. Furthermore, they embrace shared decision-making and serve as a link bringing the healthcare team, residents and their care partners together for coordinated care planning [43,54]. As a result, NPs globally can be in a better position to deliver timely and person-centered care to LTC home residents, which includes the provision of a dignified death.

Although this study provides new insights into the evidence involving the role of NPs in the integration of a palliative approach, it has limitations. It is an exploratory study involving a small sample of NPs; hence, the findings may not be transferable to other regions. However, the NPs included in our study worked in geographically diverse regions of Ontario, including both private and not-for-profit homes serving a wide range in the number of residents. In addition, we did not compare the experiences of NPs working in different models, i.e., attending or outreach and in different types of LTC homes. This was due to them all having performed similar functions and conveying the same sense of duty in caring for their residents who are experiencing the EOL. However, future research should explore the differences between the roles, responsibilities, and outcomes associated with the two groups of NPs working in LTC settings. Finally, the study did not include other LTC home staff such as nurses and administrators. This results in missing insights into their role in implementing an integrated palliative approach and supporting a dignified death to their residents during the pandemic.

## 5. Conclusions

Despite numerous challenges, the NPs played a critical role in facilitating a dignified death for LTC residents through ACP and goals of care discussions, EOL pain and symptom management and care measures after the death of residents. A dignified death for residents was accomplished in close collaboration with LTC home staff. The COVID-19 pandemic has emphasized the need to take an upstream approach in implementing an integrated palliative approach to care for residents and their care partners that focuses on the needs of the whole person while acknowledging their mortality. While LTC homes need to implement this approach, in light of the exceptional circumstances imposed by the pandemic, it is critical that interventions are in place to address complicated bereavement in care partners and moral distress in LTC home staff.

## Figures and Tables

**Table 1:** Characteristics of study participants and their workplace.

Participant Characteristics	
Average Age (range)	45 (28–66)
Gender (%)	
Men	3 (21%)
Women	11 (79%)
Years of work experience (range)	9·3 (2–21)
Group (%)	
Attending NP	8 (57%)
NP outreach team	6 (43%)
LTC home Ownership (%)	
For-profit	6 (43%)
Not-for-profit	8 (57%)
Beds in LTC homes where participants work	182 (62–302)

**Table 2:** Categories and themes related to the Nurse Practitioners’ role in facilitating a dignified death in long-term care homes during the COVID-19 pandemic using Thematic Analysis [34].

Thematic Analysis Steps	Codes, Categories and Themes
1. Familiarization with data	1.1. The full corpus of interviews was transcribed by a professional transcription service and reviewed for accuracy against the recordings by the RC. 1.2. The primary (AK) and secondary analysists (SV, AC, LY, NZ) created a list of 10 initial themes: NP Clinical Responsibilities, NP Leadership Responsibilities, NP Educational Responsibilities, NP Administrative Responsibilities, Staffing, Infection Control and Prevention, Resident Care, Assuming Multiple Roles, Pandemic Preparedness, Interprofessional Collaboration.
2. Generation of initial themes	2.1. Additional themes generated included: ACP; palliative and EOL care; virtual care; resident outcomes; death and dying; confinement and isolation. 2.2. If an analyst identified a new topic in a transcript, they would engage in discussion with other analysts to see if the topic fit into one of the previously identified themes or a new theme was required to be generated.
3. Identification of broader categories	3.1. The research team reviewed the full list of themes to identify sub-categories. For example, when participants talked about palliative care, an initial theme, they did so in the context of how they carried out ACP and goals of care conversations, so we included this theme in the sub-category, “Taking a proactive approach to facilitate mass ACP conversations”. 3.2. Upon review of sub-categories, the research team then aggregated them into three broader categories. For example, sub-categories “Taking a proactive approach to facilitate mass ACP conversations” and “Connecting with care partners for difficult yet critical discussions (goals of care)” were grouped into one category, “ACP and goals of care discussion”. 3.3. The initial themes were collated into the agreed upon categories and sub-categories by the analysis team.
4. Review of categories and consensus	4.1. The team checked each sub-category against the organized, coded data to ensure internal consistency and polished them as needed. For example, two sub-categories were merged, i.e., “Keeping a vigil at the time death” and “psychosocial needs of residents”, into a single sub-category, “Addressing psychosocial needs of residents and care partners”. 4.2. The analysis team reviewed all identified categories against the developing topics to make sure that they correctly represented the meanings manifest in the dataset as a whole. 4.3. All identified categories and sub-categories were discussed by the research team to draw mutual links between them and devise an outline that tells the story of the data.
5. Defining and naming final categories	5.1. Using a consensus approach, the research team generated names and definitions for the final categories and sub-categories listed below: A. ACP and goals of care discussion A.1. Taking a proactive approach to facilitate mass ACP conversations A.2. Connecting with care partners for difficult yet critical discussions (goals of care) B. Pain and symptom management at the EOL B1. Optimizing emergency supplies B2. Prescribing anticipatory medications to aid symptom management B3. Consulting with experts where needed B4. Addressing psychosocial needs of residents and care partners C. Care after death C1. Being present with staff for the dignified performance of last offices C2. Providing emotional support to staff and family upon death

## Data Availability

Not applicable.

## Supplementary Materials

The following are available online at https://www.mdpi.com/article/10.3390/jpm11050433/s1, Table S1: Semi-Structured Interview Guide with Nurse Practitioners.
