# Supplementary material for: Complexity of Nurse Practitioners’ Role in Facilitating a Dignified Death for Long-Term Care Home Residents during the COVID-19 Pandemic
Source: J Pers Med. 2021 May 19;11(5):433. doi: 10.3390/jpm11050433 (PMC8161387; doi:10.3390/jpm11050433)
Supplement: Supplementary file 1 [file jpm-11-00433-s001.zip › jpm-1223282-supplementary.pdf]

Supplementary Material: Table S1

Semi-Structured Interview Guide with Nurse Practitioners

1. Where do you work as a Nurse Practitioner (NP)?

(Probe: day-to-day schedule; tasks that are performed as part of usual role)

2. What was your role as an NP working in a long-term care (LTC) home before the pandemic?

(Probe: clinical, leadership, administrative responsibilities)

3. What is your opinion on the preparedness for the pandemic of the LTC homes you work at?

(Probe: processes in place to ensure preparedness for the pandemic)

4. Which successful responses by the LTC home to the pandemic did you observe?

(Probe: management preparedness, policies and procedures, training for staff, staff capacity)

5. Did you observe any gaps in pandemic preparedness?

(Probe: management preparedness, policies and procedures, training for staff, staff capacity)

6. Were there any policies, procedures, or protocols established in response to the pandemic in the home(s) that you work at?

(Probes: changes in relation to coroner's office; changes in relation to ministry inspectors)

7. Please describe your role in infection control and prevention?

(Probe: role in PPE access, use, and disposal; capacity building activities; developing isolation care plans)

8. What were your responsibilities in supporting staff during the pandemic?

(Probe: availability of emotional support; team support)

9. Please describe your role in supporting the quality of care for the residents during COVID-19?

(Probe: working with residents' families; changes in practices of activities of daily living; maintaining social connectedness; end-of-life care; advanced care planning; residents purposefully walking)

10. What were some of the challenges that interfered with your work during the pandemic?

11. In your opinion, how could these be prevented in a future pandemic?

12. What helped you in performing your role as an NP during the pandemic?
